# Is a 10-sow unit economically sustainable? A profitability assessment of productivity amongst small-holder pig farmers, Mpumalanga, South Africa

**DOI:** 10.4102/ojvr.v83i1.1011

**Published:** 2016-05-12

**Authors:** Priscilla Munzhelele, James W. Oguttu, Folorunso O. Fasina

**Affiliations:** 1 Nooitgedacht Research Station, Department of Agriculture, Rural Development, Land and Environmental Affairs, Animal Research, Non-ruminant Sub-directorate, South Africa; 2 Department of Agriculture and Animal Health, University of South Africa, South Africa; 3 Department of production Animal Studies, University of Pretoria, South Africa

## Abstract

The majority of small-holder pig farmers in Mpumalanga had between 1- and 10-sow herds. The main aim of this study is to evaluate the current government agricultural intervention (supply of 10 sows and a boar) in terms of technical and economic feasibilities and ascertain whether the small-scale pig value chain system alleviates poverty. Data were obtained from 220 randomly selected small-holder pig farmers using a semi-structured questionnaire. The results showed that 58% farrowed ≤ 10 piglets/born/sow/litter, 44.2% practiced no weaning method and many fed swill and leftovers alone (41.6%). Pair-wise association revealed that the feeding of commercial feeds had a relationship with pigs in relatively good to very good body condition. Pigs in poor body condition were positively correlated with the feeding of swill alone. The economic models for the 10-sow unit proved that pig farming is unprofitable if the current management and feeding systems that operate in the commercial industry are utilised. However, only through a combination of cooperative systems, benefits of economies of scale, reduction of preweaning mortalities and structured government inputs can pig production be profitable at this scale of production.

## Introduction

Animal agriculture in general and pig production in particular are important economic activities globally (Dietze 2011; Mokoele *et al*. 2015; Roelofse 2013). In South Africa, pig production is distributed in all nine provinces, with higher concentrations in Limpopo, North West, Gauteng and KwaZulu-Natal, partially because of cultural and religious preferences and availability of feedstuffs. Mpumalanga is placed sixth in terms of pig production, contributing approximately 8% of the national pig herd (DAFF 2012; MPG 2013). The province had a relatively high concentration of small-holder pig farmers otherwise known as emerging small-scale pig farmers. Pig farming requires little space, yields a large number of offspring after a shorter gestation period than other small stock and can be combined with other forms of subsistence agriculture where land resources are scarce (DAFF 2012; Makiwane *et al*. 2012). In addition, it plays a major role in poverty reduction and food security (FAO 2004) and provides a form of investment, emergency cash and meat for home consumption (Drucker & Anderson 2004; Mhlanga 2002). In Mpumalanga, the commonly found breeds of pigs are the Kolbroek, Large White, Landrace and their crosses.

Backyard pig farming and semi-intensive management systems in poorly designed pens are the most common small-holder pig farming practices in the rural and peri-urban areas of Mpumalanga. In general, the farm families rely on family labour and the majority of products are meant for household consumption or converted to cash for the purpose of family maintenance. These contributions are very important for family incomes in Mpumalanga, where poverty rates have ranged from 50.4% (1996), 59.1% (2004), 50.1% (2008) to 39.4% (2011) and the unemployment rate remains at 29.4% (MPG 2009, 2013).

The Mpumalanga Department of Agriculture, Rural Development, Land and Environmental Affairs (DARDLEA) established the programme called *Masibuyele esibayeni*, meaning *back to the kraal* (i.e. returning to the land), with similar programmes in Gauteng and Limpopo amongst others. The programme is aimed at helping the small-holder farmers to upgrade and boost productivity by improving the genetic pool of their livestock. For the pig component, DARDLEA provides farmers with 10 sows and 1 boar with improved genetics for breeding and provides supportive services to such farms. To evaluate the potential success of such a programme, we analysed profit determinants, the economic feasibility and viability of such small-holder projects and suggested options for improvement of the programme.

## Materials and methods

### Study area and data collection

**FIGURE 1:**
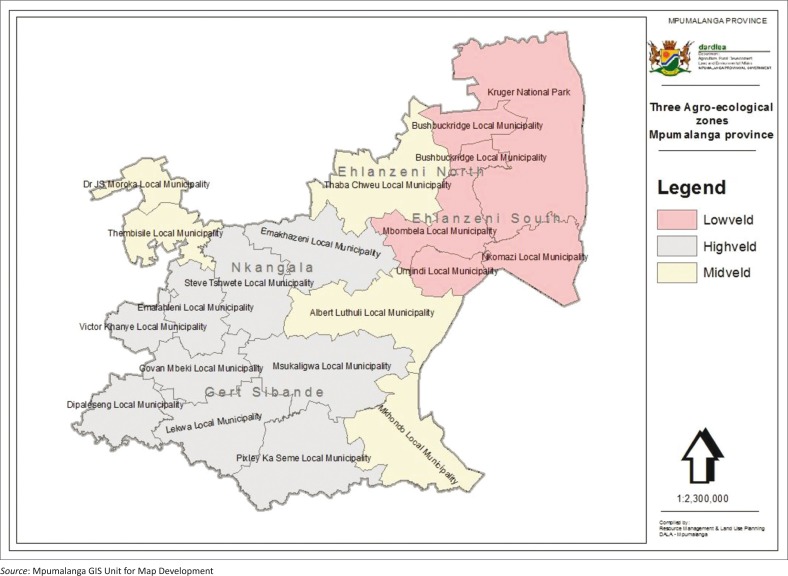
Map of Mpumalanga with demarcation of agro-ecological zones.

The study was approved by the Ethical Committee of the College of Agriculture and Environmental Sciences, UNISA (CAES) with an Ethical approval number: 2013/CAES/140. A recent document targeting small-holder farmers indicated that at least 5889 small-holder farms exist in Mpumalanga (DAFF 2013; Figure 1). We used this number as the sample frame. The sample size was calculated for frequency using the formula:

$$\begin{array}{l} \text{Sample size},n=[\text{DEFF }\times \;Np(1-p)]/\\ {}[(d^{2}/Z_{21}-\alpha /2(N-1)\\ +p(1-p)], \end{array}$$ [Eqn 1]

Where, Population size (for finite population correction factor or fpc) (*N*): 5889; hypothesised % frequency of outcome factor in the population (*p*): 50% ± 5; Confidence limits as % of 100 (absolute ± %) (*d*): 5%; Design effect (for cluster surveys-DEFF): 1.

A total of 361 farms were needed. We continued to recruit small-holder pig farms randomly until we could identify no more farms of interest. A final list of 220 farmers was generated from the list provided by DARDLEA and the additions were made through consultations with farmers, extension officers, animal health technicians and community leaders. All identified farmers were visited and data were collected through the use of a semi-structured pretested questionnaire. Direct observations were evaluated through a checklist, and photographic documentation was obtained, where necessary.

The services of the extension officers and animal health technicians from DARDLEA, who were previously trained on questionnaire administration, were employed. The inclusion criteria were as follows: (1) ownership of ≥ 1 to ≤ 50 pigs and (2) resident within the province and active in the small-holder industry. The English questionnaire was translated and administered using local home languages (Zulu, IsiNdebele, Shangaan and IsiSwati) for the understanding of the study participants.

### Statistical analyses and management

All responses were entered into Microsoft Excel 2007^®^ spreadsheet and filtered. Data were analysed using Stata v9 (Statacorp., Texas, USA) and hypotheses were tested using appropriate analytical methods. To determine associations, all data were re-entered as 1 = yes and 0 = no and coded correctly for the Stata programme. Using Pearson’s Chi-square test, outputs were generated to associate certain variables and preferred methods, including markets, market determinants, treatment methods for sick pigs, feed preference, body conditions of the sows and age at weaning.

To integrate economic analyses, a partial budgeting and return-on-investment (ROI) model was developed in a Microsoft Excel 2007^®^ spreadsheet. Outcomes from the data obtained, including details from the field and published materials, were used to develop and validate the model. Economic feasibility and viability of a 10-sow unit was tested for a 3-year farm operation. Details of the inputs and outputs are available in the Appendix. The sensitivity analyses were tested by varying some parameters, including the reduction in feed price, removal of farmer’s remuneration, transport cost and reduction or preweaning deaths. Outputs were generated in tables and graphs, and the model is freely available in excel format for the use of small-holder farmers and development partners (see Appendix).

## Results

### Descriptive statistics

Approximately 41% of the farms surveyed confirmed that the sows farrowed ≥ 11 piglets per litter and more than 58% farrowed ≤ 10/sow/litter. Only about 19% weaned at 1 month (industry standard) and only 11% depended on commercial feed completely (Table 1). The majority of the farmers mixed commercial feed with swill (41.6%) or fed swill and leftovers alone (47%), and about 69% resorted to home medication, allowed the animal to die or sent any sick animal for slaughter (Table 1). Only 27% sold their porkers at less than 6 months, whereas the majority (73%) marketed their pigs above 7 months. Less than 10% of all sows were in adequate body condition (at least a score of 3). The prevailing local and market prices were the main determinant for marketing pigs and incomes arising from the sale were used mainly in the home or to maintain the remaining pigs (Table 1).

**TABLE 1:** Profit and market-related variables of small-holder farmers, Mpumalanga.

Variables	Descriptors	Mpumalanga, 95% CI	*p*-value
Average numbers of piglets farrowed/sow/litter (*n* = 217)	1–5	9.7 (5.7, 13.7)	< 0.0001
	6–10	48.6 (41.9, 55.3)	
	11–15	35.2 (28.8, 41.6)	
	16–20	6.5 (3.2, 9.8)	
Weaning age (*n* = 217)	1 month	19.4 (14.1, 24.7)	< 0.0001
	2 months	22.6 (17.0, 28.2)	
	3 months	13.8 (9.2, 18.5)	
	No weaning	44.2 (37.6, 50.9)	
Feed fed to pigs (*n* = 219)	Buy feeds	11.4 (7.2, 15.7)	< 0.0001
	Buy feeds and supplement	41.6 (35.0, 48.1)	
	Leftovers	47.0 (40.4, 53.7)	
What do you do when pigs are sick? (*n* = 219)	Ethno-veterinary preparations†	13.2 (9.3, 18.4)	< 0.0001
	Home medication‡	33.8 (27.9, 40.3)	
	Consult professionals	17.4 (12.9, 23.0)	
	Leave to die or slaughter	35.6 (29.6, 42.2)	
Age at which pigs are sold (*n* = 217)	Less than 6 months	26.7 (21.3, 33)	< 0.0001
	7 months to 18 months	61.8 (55.1, 68.0)	
	Above 19 months	11.5 (7.9, 16.5)	
Body condition of sows (*n* = 219)	Very poor to poor (≤ 2)	41.1 (34.5, 47.7)	< 0.0001
	Poor to good (2.5–3)	50.2 (43.6, 56.9)	
	Good to very good (3–3.5)	7.8 (4.2, 11.3)	
	Very good to obese (3.5–4)	0.9 (-0.4, 2.2)	
Price determinant (*n* = 215)	Age of the pig	22.3 (17.3, 28.4)	< 0.0001
	Gender of the pig	4.2 (2.1, 7.9)	
	Prevailing local price	57.2 (50.5, 63.6)	
	Prevailing market price	55.8 (49.1, 62.3)	
Preferred market (*n* = 217)	Abattoir	8.3 (5.2, 12.8)	< 0.0001
	Auction	51.2 (44.5, 57.7)	
	Local slaughters/sold live	64.0 (57, 70.0)	
Uses of income from pig sales (*n* = 217)	Home groceries	64.5 (57.9, 70.6)	< 0.0001
	Education	30.4 (24.7, 36.8)	
	Maintenance of family	27.2 (21.7, 33.5)	
	Maintenance of pigs	66.0 (59.4, 71.9)	
	Others	5.1 (2.8, 8.9)	

† Ethno-veterinary preparations used by the small-holder farmers include aloe, blue bar soap, potassium manganate, used engine oil, salt and feed, and used vegetable oil.

‡ Home medication includes the inappropriate use of antibiotics and antihelmithics like ivermectin and others.

Using the Pearson’s Chi–square test, the prevailing market price significantly influenced the preference for abattoirs (*χ*^2^ = 8.96, *p* < 0.005), auctions (*χ*^2^ = 135.51, *p* < 0.0001) and local slaughter slabs (χ^2^ = 72.71, *p* < 0.0001) as a means of disposal of final products (Table 2). Similarly, the prevailing local price of product had an influence on sales at auctions (χ^2^ = 39.74, *p* < 0.0001) and local slaughter slabs (χ^2^ = 114.39, *p* < 0.0001). Age of pigs at sale slightly influenced the sale at auctions (χ^2^ = 6.11, *p* = 0.01), but significantly influenced sales at local slaughter slabs (χ^2^ = 28.97, *p* < 0.0001). There was a significant association between the use of ethno-veterinary preparations and sales at auctions (χ^2^ = 11.37, *p* = 0.001) or at local slaughter slabs (χ^2^ = 7.30, *p* < 0.01). Furthermore, farmers who medicated their pigs themselves disposed off their products at the auctions (χ^2^ = 6.87, *p* < 0.01) or at the local slaughter slabs (χ^2^ = 11.35, *p* = 0.001) (Table 2). Finally, only the local slaughter slabs and the slaughter of sick animals were associated (χ^2^ = 6.58, *p* = 0.01).

**TABLE 2:** Association between preferred methods of marketing, market price determinants and type of treatment for sick animals.

Investigated factor	Variable	Methods of marketing					

		Abattoir		Auction		Local slaughter	
		
		χ^2^	*p*	χ^2^	*p*	χ^2^	*p*
Market price determinants	Age of pig	0.0001	0.99	6.11	0.01	28.97	< 0.0001
	Gender of pig	0.0979	0.75	3.15	0.08	2.76	0.10
	Prevailing local price	4.73	< 0.05	39.74	< 0.0001	114.39	< 0.0001
	Prevailing market-related price	8.96	< 0.005	135.51	< 0.0001	72.72	< 0.0001
Type of treatment for sick animals	Ethno-veterinary preparations†	0.94	0.33	11.37	0.001	7.3	< 0.01
	Home medication	2.21	0.14	6.87	< 0.01	11.35	0.001
	Consultation veterinary professional‡	0.3	0.58	0.08	0.36	1.99	0.16
	Slaughter the sick animal	1.51	0.22	0.92	0.34	6.58	0.01

The *p*-values are indicated in parentheses.

† Ethno-veterinary preparations was indicated in footnote to Table 1.

‡ Consult vets means consultation with private or government veterinarians including veterinary para-professionals.

There were associations between the feeding of commercial rations solely and poor body condition (χ^2^ = 9.75, *p* < 0.005) and poor–fairly good condition (χ^2^ = 5.46, *p* < 0.05). Farmers who weaned their piglets at 1 month had their sows in very good body condition comparatively (χ^2^ = 8.55, *p* < 0.005), whilst those who weaned at about 3 months had good–very good body condition (χ^2^ = 6.46, *p* = 0.01) and those who did not wean at all had their sows in poor (χ^2^ = 8.80, *p* < 0.005) or good–very good condition (χ^2^ = 11.56, *p* = 0.001) (Table 3). Similarly, there were associations between weaning at 1 month and feeding of commercial ration (χ^2^ = 19.80, *p* < 0.0001), mixing of commercial ration and swill (χ^2^ = 11.47, *p* = 0.001) and mixing of swill and household remnants alone (χ^2^ = 10.62, *p* = 0.001) (Table 4).

**TABLE 3:** Association between body condition scores, types of feed used and age at weaning.

Investigated factor	Variable	Body conditions							

		Poor (≤2)		Poor–Good (2.5–3)		Good–very good (3–3.5)		Very good–Obese (3.5–4)	
			
		χ^2^	*p*	χ^2^	*p*	χ^2^	*p*	χ^2^	*p*
Feed types	Only commercial ration	9.8	< 0.005	5.5	< 0.05	2.29	0.13	3	< 0.10
	Commercial ration and swill	3.1	< 0.10	1.8	0.18	0.55	0.46	1.8	0.18
	Only swill and remnants	3	< 0.10	1.2	0.28	0.44	0.51	1.7	0.18
Age at weaning	1 month	3.3	< 0.10	0.5	0.49	2.57	0.11	8.6	0.003
	2 months	0.5	0.50	0.2	0.63	0.34	0.56	0.6	0.45
	3 months	0.8	0.36	0.2	0.69	6.46	0.01	0.3	0.57
	No weaning	8.8	< 0.005	0.7	0.42	11.6	0.001	1.6	0.21

The *p*-values are indicated in parentheses. Swill refers to kitchen swill, restaurant swill, hospital swill, school swill, ripe fruit, cow’s milk, maize, vegetables.

**TABLE 4:** Association between types of feed used and weaning age.

Investigated factor	Variable	Types of feed used					

		Only commercial ration		Commercial ration and swill		Only swill and remnants	
		
		χ^2^	*p*	χ^2^	*p*	χ^2^	*p*
Age at weaning	1 month	19.78	< 0.0001	11.47	0.001	10.62	0.001
	2 months	0.64	0.42	0.07	0.79	0.17	0.68
	3 months	2.22	0.14	0.51	0.47	0.68	0.41
	No weaning	2.80	0.09	3.25	< 0.10	3.13	< 0.10

The *p*-values are indicated in parentheses.

Significant association existed between production of a larger number of piglets and feeding of commercial ration (χ^2^ = 11.57, *p* = 0.001). In contrast, the mixing of swill and commercial ration was associated with the production of low (χ^2^ = 17.25, *p* < 0.0001) to medium (χ^2^ = 23.11, *p* < 0.0001) numbers of piglets per litter (Table 5). The feeding of swill only produced similar significant results (Table 5). Similarly, sows in poor body condition produced low (χ^2^ = 6.37, *p* = 0.01) to medium numbers of piglets per litter (χ^2^ = 5.44, *p* = 0.02) (Table 5). Only sows in very good body condition were associated with a large number of piglets per litter (χ^2^ = 7.77, *p* = 0.005).

**TABLE 5:** Association between average number of piglets farrowed per sow per litter, types of feed used and body condition scores.

Investigated factor	Variable	Average number of piglets farrowed per sow per litter							

		1–5 piglets		6–10 piglets		11–15 piglets		≥ 16 piglets	
			
		χ^2^	*p*	χ^2^	*p*	χ^2^	*p*	χ^2^	*p*
Feed types	Only commercial ration	0.08	0.78	2.8	< 0.10	0.37	0.54	11.57	0.001
	Commercial ration and swill	1.99	0.16	17.25	< 0.0001	23.11	< 0.0001	0.99	0.32
	Only swill and remnants	1.08	0.30	19.51	< 0.0001	21.31	< 0.0001	0.87	0.35
Body conditions	Poor (≤ 2)	6.37	0.01	4.88	< 0.05	5.44	< 0.05	3.09	< 0.10
	Poor–Good (2.5–3)	4.26	< 0.05	0.89	0.35	3.94	0.05	0.35	0.55
	Good–very good (3–3.5)	0.36	0.55	1.63	0.20	0.16	0.69	1.22	0.27
	Very good–obese (3.5–4)	0.21	0.64	1.84	0.18	0.21	0.64	7.77	0.005

The *p*-values are indicated in parentheses.

### Economic models

**FIGURE 2:**
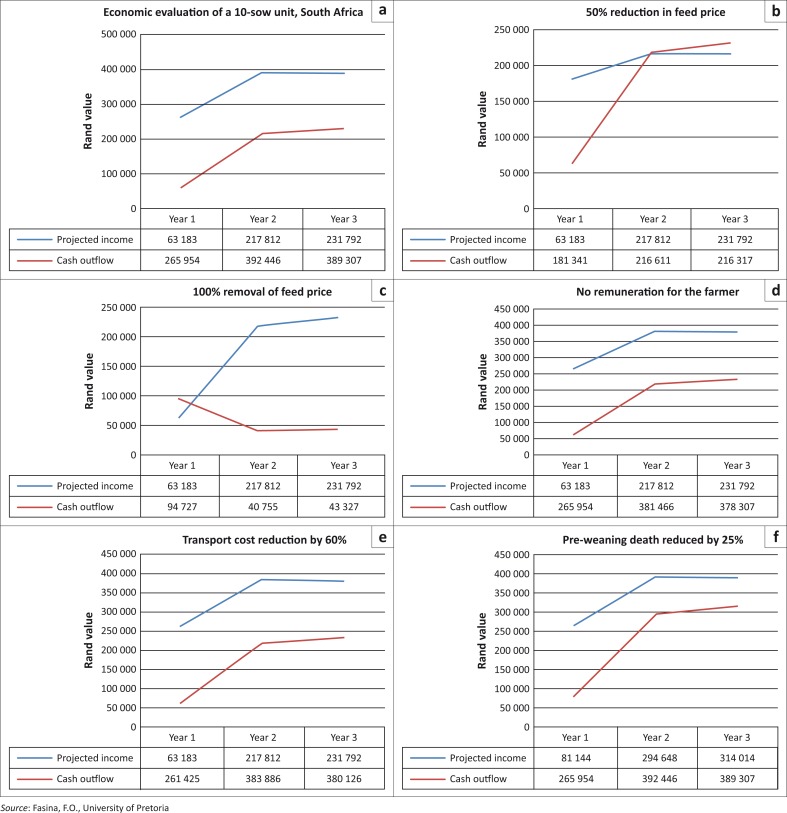
Economic evaluation of a 10-sow unit using different scenarios. (a) Economic evaluation of a 10-sow unit, South Africa, (b) 50% reduction in feed price, (c) 100% removal of feed price, (d) No remuneration for the farmer, (e) transport cost reduction by 60% and (f) preweaning death reduced by 25%.

Using partial budgeting and ROI models, the 10-sow unit pig farm continues to utilise more cash (outflow) than the receipts that came into the farm account. Feed (using commercial ration) accounted for at least 75% of the annual cash outflow for any 1 year (Figure 2a; Table 1-A1, Table 2-A1, Table 3-A1). With a 50% reduction in feed price through supplementation with swill and leftovers from the home, the model became economically viable towards the end of the third year of operation (Figure 2b). However, a 100% reduction in feed price through complete replacement with swill would make the farm model break even at the beginning of the second year of operation with subsequent profits (Figure 2c). With complete removal of remuneration for the farmer over a 3-year project cycle, the farm was still economically unsustainable (Figure 2d) and similar results were obtained with a 60% reduction in transport cost (Figure 2e) and improving the farm productivity through a 25% reduction in preweaning mortalities (Figure 2f).

## Discussion

In Mpumalanga, there are at least 15 auction facilities that are randomly dispersed. Many of the small-holder farmers marketed their pigs at auctions or within the communities. Because strong association existed between auctions and prevailing market price, it can be inferred that high pig populations at auctions are indications that the prevailing market prices are good. As such, market price is a driver for moving pigs to auctions. Such pigs often evade ante- and postmortem inspections and may inadvertently spread infectious diseases. It becomes necessary to identify each auction within Mpumalanga and know the farms and road networks that support them so as to plan and apply intervention strategies where and when necessary, for example, in the case of a rapidly spreading animal disease or for surveillance purposes. Secondly, market price had some degree of influence in moving pigs to the abattoirs but only a minority (8.3%) preferred this option for marketing. Similar results have been reported from Limpopo, where farmers travelled a long distance to obtain higher prices primarily at auctions and at abattoirs (Mokoele *et al*. 2014).

Furthermore, because of the association that exists between local price and local markets (Table 2), we inferred that the local market is highly influenced by prevailing local price, with minor influence from the age and sex of the pig. Primarily, sick, unthrifty pigs, mature boars and late-maturing pigs are slaughtered locally and according to our results, only the local slaughter slabs were associated with the slaughter of sick animals. Comparatively, prevailing market prices are higher than the local price range and better quality products are often sent to the auctions (51%) and commercial abattoirs (8.3%). It is known that pork from mature boars has ‘boar taint’, a pheromonal smell (androstenone and skatole), and generally attracts much lower prices compared with other pork. In addition, slow-growing, late-maturing pigs are characteristic of most small-holder pig producers, an indication that they will be presented for slaughter at later age. It is therefore not surprising that local market and age of pigs are associated.

Although a positive association exists between auctions and local slaughter slabs and home medication and ethno-veterinary usages, similar qualitative evaluations have revealed that small-holder pig farmers who sell at auction and locally tend to medicate the pigs themselves with ethno-veterinary preparations and also to use, sometimes incorrectly, long-acting oxytetracycline and ivermectin (Fasina *et al*. 2012; Mokoele *et al*. 2014). Although DARDLEA has public veterinarians and animal health technicians in all the districts and municipalities within the province who provide free services to small-holder farmers, it appeared that small-holder pig farmers lacked information about veterinary services in the province, or intentionally refused to seek veterinary assistance; these farmers hardly consult veterinarians and para-veterinary professionals (Table 2). Reasons for this disconnect must be established and a drive to gain small-holder farmers’ trust and to make service accessible and affordable must be implemented.

Local slaughter for household consumption and sale within the community was prevalent in our study group (64%). This poses a high risk to animal and public health because preslaughter pig inspections are neglected, as highlighted above. Whereas local slaughter for community sale is prohibited by law, slaughter for household consumptions is allowed under South African law (Anonymous 2000 [*Meat Safety Act* no. 40 of 2000]).

There is an insignificant degree of association between auctions and sex of animals and the reason for this observation is evident. Top quality breeding boars are priced beyond the reach of small-holder farmers and good commercial boars are equally expensive. As such, the small-holder farms often settle for lower quality boars sourced from auctions. This practice exposes the small-holder farms to risks of infectious animal diseases and the genetic value of the boars is doubtful. It will be important for agricultural authorities to revise their strategies, evaluate these gaps and devise means of addressing them. A good interim measure may be the creation of district and municipal pig-breeding centres to multiply quality genetics and distribute them to small-holder farmers at minimum costs. Such an intervention would carry additional benefits of employment generation and provision of training in animal production, animal health and biosecurity.

Although it was established that the feeding of commercial rations alone is significantly associated only with poor to fairly good body condition, it was also established that such feeds are often rationed and pigs were underfed because of cost. However, results showed that well-conditioned animals tend to produce larger numbers of piglets per litter and early weaning was positively associated with good condition across the panel of feeding. The combination of good quality commercial ration supplemented with risk-free cooked swill may offer alternative feeding strategies in small-scale pig production.

Using partial budgeting and ROI, the 10-sow unit pig farm is economically unsustainable with commercial feed. It appeared that the main driver of profitability in small-holder pig farms is the feed cost. Whereas the profitability of a pig production unit increases with an increase in the number of live-born piglets per litter (Kyriazakis & Whittemore 2006), our results only partially agreed with this assertion. Even when the production efficiency increased and preweaning mortality was reduced by 25%, a 10-sow pig production unit was still not able to break even in this analysis.

Feed is a pig farm input that is indisputably of utmost importance (Kyriazakis & Whittemore 2006); our analyses have confirmed this, as it was the most important determinant of profitability in small-holder farms. Although using commercially compounded ration by farmers should yield better quality products and improve reproduction and overall production, at this scale of production it was not financially feasible and viable. Previous studies have highlighted some of the reasons for the relatively high feed cost and suggested reasons why small-holder pig farmers rely on swill as an alternative form of feed (Phengsavanh *et al*. 2010; Roelofse 2013). We have similarly concluded that because of the infeasibility of feeding commercial ration, small-holder farmers will continue to feed swill and alternative feed sources for the unforeseeable future.

We are aware that it is potentially possible for small-holder pig farmers to make profit (Lapar & Staal 2010; Petrus *et al*. 2011; Phengsavanh *et al*. 2011) and have demonstrated that in this economic analysis. However, the ≤ 10-sow unit, small-holder farmers in Mpumalanga were able to achieve profitability mainly through the use of swill as a major source of feed. This practice is common to most small-holder pig farms elsewhere in South Africa (Gcumisa 2013; Roelofse 2013). The feeding of swill comes with potential risk of spread of diseases to pigs (e.g. salmonellosis, campylobacteriosis, African swine fever, classical swine fever, porcine reproductive and respiratory syndrome and foot and mouth disease) and possible transmission of zoonotic diseases from pigs to humans (Beltrán-Alcrudo *et al*. 2008; DAFF 2005; Haynes 2001). In addition, pig products originating from swill feeding may not reach the quality required by South African pig abattoirs.

In view of the above risks and knowing that it is more realistic for small-holder farmers to make a profit with units of between 50 and 100 sows (Roelofse 2013), we suggest that the agricultural authorities should assist farmers in the development of community self-help groups and farmers’ cooperatives. Such calls have been made previously (Munyai 2012) and the successes associated with such organisations by small-holder pig farmers have been documented in Namibia (Petrus *et al*. 2011), Vietnam (Lapar & Staal 2010) and Lao People’s Democratic Republic (Phengsavanh *et al*. 2011). Such cooperative organisations have the advantages of bulk purchase of feed with benefits of economies of scale and discounts (Costales *et al*. 2007; Lapar & Staal 2010), reduced transport tariffs through bulk transport and better negotiating power.

In addition, because an improvement in efficiency and reduction of preweaning loss by about 25% will improve profitability, it is necessary to implement the measures required to achieve these objectives. The government may also consider tax rebates on animal feed products that are directed to small-holder pig farmers. Only through the combination of the above measures and interventions will small-holder farmers with ≤ 10-sow units be able to break even and use pig production as a means of poverty alleviation.

## Conclusion

Small-holder pig production and health management will continue to be relevant in an emerging economy like South Africa. However, for government agricultural interventions to provide the desired benefits, empirical evaluations for technical and economic feasibilities must be carried out. Although a 10-sow unit is technically feasible, in Mpumalanga and elsewhere in South Africa, the current input systems negate the benefits that should come with such programmes. Proposed models and revisions as suggested above may facilitate government interventions and make pig production more attractive to small-holder farmers.

## Appendix 1

**TABLE 1-A1:** Project cash flow statement for a model 10-sow unit, Mpumalanga, 2015 (year 1).

Description	Jan.	Feb.	Mar.	Apr.	May	June	July	Aug.	Sept.	Oct.	Nov.	Dec.	Total	Source
Opening bank balance	1500	0	0	0	0	0	0	0	0	0	0	0	1500	Department of Labour (2015)
Project income (invoiced amount)	0	1500	1500	1500	1500	1500	1500	1500	1500	17 061	17 061	17 061	63 183	ABSA (2015); Fasina *et al*. (2012); Munzhelele, Oguttu & Fasina, unpublished data
Loan	0	0	0	0	0	0	0	0	0	0	0	0	0	-
**Total cash inflow**	**1500**	**1500**	**1500**	**1500**	**1500**	**1500**	**1500**	**1500**	**1500**	**17 061**	**17 061**	**17 061**	-	-
**Once-off costs**	**59 000**	-	-	-	-	-	-	-	-	-	-	-	-	-
Building material	**30 000**	-	-	-	-	-	-	-	-	-	-	-	30 000	Author’s estimate
10 Sows @ R2500	**25 000**	-	-	-	-	-	-	-	-	-	-	-	25 000	Auction price
1 Boars @ R4000	**4000**	-	-	-	-	-	-	-	-	-	-	-	4000	Auction price
Farmer’s remuneration	0	0	0	0	0	0	0	0	0	1000	1000	1000	3000	Opinion survey
Accounting fees	0	0	0	0	0	0	0	0	0	0	0	0	0	-
Bank charges	0	100	100	100	100	100	100	100	100	100	100	100	1100	Author’s estimate
Disinfectants	0	0	0	0	0	0	0	0	0	0	0	0	0	-
**Feed (48 tonnes/annum)**	**0**	**2996**	**2996**	**2996**	**2996**	**11 833**	**29 882**	**29 882**	**29 882**	**29 882**	**29 882**	**29 882**	203 109	Fasina *et al*. (2012), Munzhelele, Oguttu & Fasina, unpublished data
10 Sows + 1 boar	0	2996	2996	2996	2996	2996	2996	2996	2996	2996	2996	2996	32 956	Roelofse (2013); Authors’ calculations
Weaners	0	-	-	-	-	8837	8837	8837	8837	8837	8837	8837	61 859	Authors’ calculations
Growers, porkers and finishers	0	-	-	-	-	-	18 049	18 049	18 049	18 049	18 049	18 049	108 294	Authors’ calculations
Breeders	-	-	-	-	-	-	-	0	0	0	0	0	0	-
Others	0	-	-	-	-	-	-	-	0	0	0	0	0	-
Labour costs	0	0	0	0	0	0	0	0	0	250	250	250	750	Opinion survey
Medicines and vaccines	485	485	485	485	485	485	485	485	485	485	485	485	5820	Fasina *et al*. (2012)
Telephone	0	0	0	0	0	0	0	0	0	0	0	0	0	-
Transport	0	3627	-	-	-	-	-	-	-	3627	3627	3627	14 508	Author’s calculations
Water and electricity	0	450	450	450	450	450	450	450	450	450	450	450	4950	Author’s estimate
Miscellaneous expenses	-	-	1279	1279	1279	1279	1279	1279	1279	1279	1279	1279	-	-
**Loan repayments**	**0**	**0**	**0**	**0**	**0**	**0**	**0**	**0**	**0**	**0**	**0**	**0**	**0**	-
Initiation fee	**0**	-	**-**	**-**	**-**	**-**	**-**	**-**	**-**	**-**	**-**	**-**	0	-
Repayment – capital and interest	**-**	**-**	**-**	**-**	**-**	**-**	**-**	**-**	**-**	**0**	**0**	-	0	-

**Total cash outflow**	**59 485**	**7658**	**5310**	-	**5310**	**14 147**	**32 196**	**32 196**	**32 196**	**37 073**	**37 073**	**37 073**	**267 954**	-

Model small-holder pig farm, Year 1 estimates; Project Cash Flow Statement for a model 10-sow unit, Mpumalanga, 2015.

Note: Please see the full reference list of the article, Munzhelele, P., Oguttu, J.W. & Fasina, F.O., 2016, ‘Is 10-sow unit economically sustainable? A profitability assessment of productivity amongst small-holder pig farmers, Mpumalanga, South Africa’, *Onderstepoort Journal of Veterinary Research* 83(1), a1011. http://dx.doi.org/10.4102/ojvr.v83i1.1011, for more information.

The bold figures only indicate important indicators. All the unbold details only contributed to the bold values.

**TABLE 2-A1:** Project cash flow statement for a model 10-sow unit, Mpumalanga, 2016 (year 2).

Description	Jan.	Feb.	Mar.	Apr.	May	June	July	Aug.	Sept.	Oct.	Nov.	Dec.	Total
Opening bank balance	−240 344	−257 872	−275 399	−292 927	−310 455	−327 983	−345 510	−363 038	−380 566	−398 094	−415 621	−433 149	−4 040 959
Project income (Invoiced amount + monthly subsidy from salary)	18 151	18 151	18 151	18 151	18 151	18 151	18 151	18 151	18 151	18 151	18 151	18 151	217 812
XYZ loan	-	-	-	-	-	-	-	-	-	-	-	-	0
**Total cash inflow**	**−222 193**	**−239 721**	**−257 248**	**−274 776**	**−292 304**	**−309 832**	**−327 359**	**−344 887**	**−362 415**	**−379 943**	**−397 470**	**−414 998**	**−3 823 147**
Owner’s remuneration	1000	1000	1000	1000	1000	1000	1000	1000	1000	1000	1000	1000	12 000
Accounting fees	0	0	0	0	0	0	0	0	0	0	0	0	0
Bank charges	100	100	100	100	100	100	100	100	100	100	100	100	1200
Disinfectants	0	0	0	0	0	0	0	0	0	0	0	0	0
**Feed**	**31 974**	**31 974**	**31 974**	**31 974**	**31 974**	**31 974**	**31 974**	**31 974**	**31 974**	**31 974**	**31 974**	**31 974**	**383 685**
10 Sows + 1 boar	3206	3206	3206	3206	3206	3206	3206	3206	3206	3206	3206	3206	38 469
Weaners	9456	9456	9456	9456	9456	9456	9456	9456	9456	9456	9456	9456	113 467
Growers, porkers and finishers	19 312	19 312	19 312	19 312	19 312	19 312	19 312	19 312	19 312	19 312	19 312	19 312	23 749
Breeders	-	-	-	-	-	-	-	-	-	-	-	-	0
Others	-	-	-	-	-	-	-	-	-	-	-	-	0
Labour costs	268	268	268	268	268	268	268	268	268	268	268	268	3216
Medicines and vaccines	519	519	519	519	519	519	519	519	519	519	519	519	6228
Telephone	-	-	-	-	-	-	-	-	-	-	-	-	0
Transport	1300	1300	1300	1300	1300	1300	1300	1300	1300	1300	1300	1300	15 600
Water and electricity	518	518	518	518	518	518	518	518	518	518	518	518	6216
**Loan repayments**	**0**	**0**	**0**	**0**	**0**	**0**	**0**	**0**	**0**	**0**	**0**	**0**	0
Initiation fee	-	-	-	-	-	-	-	-	-	-	-	-	**0**
Repayment – capital and interest	**0**	**0**	**0**	**0**	**0**	**0**	**0**	**0**	**0**	**0**	**0**	**0**	**0**

**Total cash outflow**	**35 679**	**35 679**	**35 679**	**35 679**	**35 679**	**35 679**	**35 679**	**35 679**	**35 679**	**35 679**	**35 679**	**35 679**	**392 466**

**Net cash flow**	**−257 872**	**−275 399**	**−292 927**	**−310 455**	**−327 983**	**−345 510**	**−363 038**	**−380 566**	**−398 094**	**−415 621**	**−433 149**	**−450 677**	**−4 251 292**

Note: Model small-holder pig farm, Year 2 estimates; Project Cash Flow Statement for a model 10-sow unit, Mpumalanga, 2016.

The bold figures only indicate important indicators. All the unbold details only contributed to the bold values.

**TABLE 3-A1:** Project cash flow statement for a model 10-sow unit, Mpumalanga, 2017 (year 3).

Description	Jan.	Feb.	Mar.	Apr.	May	June	July	Aug.	Sept.	Oct.	Nov.	Dec.	Total
Opening bank balance	−450 677	−469 512	−488 347	−507 182	−526 017	−544 852	−563 687	−582 522	−601 357	−610 074	−618 791	−627 508	−6 590 523
Project income (invoiced amount)	19 316	19 316	19 316	19 316	19 316	19 316	19 316	19 316	19 316	19 316	19 316	19 316	231 792
XYZ loan	-	-	-	-	-	-	-	-	-	-	-	-	0
**Total cash inflow**	**−431 361**	**−450 196**	**−469 031**	**−487 866**	**−506 701**	**−525 536**	**−544 371**	**−563 206**	**−582 041**	**−590 758**	**−599 475**	**−608 192**	**−6 358 731**
Owner’s remuneration	1 000	1000	1000	1000	1000	1000	1000	1000	1000	1000	1000	1000	12 000
Accounting fees	0	0	0	0	0	0	0	0	0	0	0	0	0
Bank charges	110	110	110	110	110	110	110	110	110	110	110	110	1320
Disinfectants	0	0	0	0	0	0	0	0	0	0	0	0	0
**Feed**	**34 212**	**34 212**	**34 212**	**34 212**	**34 212**	**34 212**	**34 212**	**34 212**	**24 094**	**24 094**	**24 094**	**24 094**	**-**
10 Sows + 1 boar	3430	3430	3430	3430	3430	3430	3430	3430	3430	3430	3430	3430	41 165
Weaners	10 118	10 118	10 118	10 118	10 118	10 118	10 118	10 118	-	-	-	-	80 943
Growers, porkers and finishers	20 664	20 664	20 664	20 664	20 664	20 664	20 664	20 664	20 664	20 664	20 664	20 664	247 966
Breeders	-	-	-	-	-	-	-	-	-	-	-	-	0
Others	-	-	-	-	-	-	-	-	-	-	-	-	0
Labour costs	287	287	287	287	287	287	287	287	287	287	287	287	3441
Medicines and vaccines	555	555	555	555	555	555	555	555	555	555	555	555	6664
Telephone	0	0	0	0	0	0	0	0	0	0	0	0	0
Transport	1391	1391	1391	1391	1391	1391	1391	1391	1391	1391	1391	1391	16 692
Water and electricity	596	596	596	596	596	596	596	596	596	596	596	596	7148
**Loan repayments**	**0**	**0**	**0**	**0**	**0**	**0**	**0**	**0**	**0**	**0**	**0**	**0**	0
Initiation fee	-	-	-	-	-	-	-	-	-	-	-	-	**0**
Repayment – capital and interest	**0**	**0**	**0**	**0**	**0**	**0**	**0**	**0**	**0**	**0**	**0**	**0**	**0**

**Total cash outflow**	**38 151**	**38 151**	**38 151**	**38 151**	**38 151**	**38 151**	**38 151**	**38 151**	**28 033**	**28 033**	**28 033**	**28 033**	**389 307**

**Net cash flow**	**−469 512**	**−488 347**	**−507 182**	**−526 017**	**−544 852**	**−563 687**	**−582 522**	**−601 357**	**−610 074**	**−618 791**	**−627 508**	**−636 225**	**−6 776 071**

Note: Model small-holder pig farm, Year 3 estimates. Project cash flow statement for a model 10-sow unit, Mpumalanga, 2017.

The bold figures only indicate important indicators. All the unbold details only contributed to the bold values.
